# Emergence of Lamivudine-Resistant HBV during Antiretroviral Therapy Including Lamivudine for Patients Coinfected with HIV and HBV in China

**DOI:** 10.1371/journal.pone.0134539

**Published:** 2015-08-19

**Authors:** Lijun Gu, Yang Han, Yijia Li, Ting Zhu, Xiaojing Song, Ying Huang, Feifei Yang, Shuo Guan, Jing Xie, Jin Gohda, Noriaki Hosoya, Ai Kawana-Tachikawa, Wenjun Liu, George Fu Gao, Aikichi Iwamoto, Taisheng Li, Takaomi Ishida

**Affiliations:** 1 China-Japan Joint Laboratory of Molecular Immunology & Molecular Microbiology, Institute of Microbiology, Chinese Academy of Sciences, Beijing, P.R. China; 2 Research Center for Asian Infectious Diseases, Institute of Medical Science, University of Tokyo, Tokyo, Japan; 3 Department of Infectious Diseases, Peking Union Medical College Hospital, Chinese Academy of Medical Sciences, Beijing, P.R. China; 4 Research Center for Asian Infectious Diseases, Institute of Medical Science, University of Tokyo, Tokyo, Japan; 5 Division of Infectious Diseases, Advanced Clinical Research Center, Institute of Medical Science, University of Tokyo, Tokyo, Japan; 6 CAS Key Laboratory of Pathogenic Microbiology and Immunology, Institute of Microbiology, Chinese Academy of Sciences, Beijing, P.R. China; University of Ottawa, CANADA

## Abstract

In China, HIV-1-infected patients typically receive antiretroviral therapy (ART) that includes lamivudine (3TC) as a reverse-transcriptase inhibitor (RTI) (ART-3TC). Previous studies from certain developed countries have shown that, in ART-3TC, 3TC-resistant HBV progressively emerges at an annual rate of 15–20% in patients coinfected with HIV-1 and HBV. This scenario in China warrants investigation because >10% of all HIV-infected patients in China are HBV carriers. We measured the occurrence of 3TC-resistant HBV during ART-3TC for HIV-HBV coinfection and also tested the effect of tenofovir disoproxil fumarate (TDF) used as an additional RTI (ART-3TC/TDF) in a cohort study in China. We obtained 200 plasma samples collected from 50 Chinese patients coinfected with HIV-1 and HBV (positive for hepatitis B surface antigen) and examined them for the prevalence of 3TC-resistant HBV by directly sequencing PCR products that covered the HBV reverse-transcriptase gene. We divided the patients into ART-3TC and ART-3TC/TDF groups and compared the efficacy of treatment and incidence of drug-resistance mutation between the groups. HIV RNA and HBV DNA loads drastically decreased in both ART-3TC and ART-3TC/TDF groups. In the ART-3TC group, HBV breakthrough or insufficient suppression of HBV DNA loads was observed in 20% (10/50) of the patients after 96-week treatment, and 8 of these patients harbored 3TC-resistant mutants. By contrast, neither HBV breakthrough nor treatment failure was recorded in the ART-3TC/TDF group. All of the 3TC-resistant HBV mutants emerged from the cases in which HBV DNA loads were high at baseline. Our results clearly demonstrated that ART-3TC is associated with the emergence of 3TC-resistant HBV in patients coinfected with HIV-1 and HBV and that ART-3TC/TDF reduces HBV DNA loads to an undetectable level. These findings support the use of TDF-based treatment regimens for patients coinfected with HIV-1 and HBV.

## Introduction

In 2011, the World Health Organization estimated that about 34 million people worldwide were infected with HIV-1, of whom approximately 12% (4 million) had chronic hepatitis B virus (HBV) infection (defined by positivity of hepatitis B surface antigen (HBsAg) in blood for >6 months) [1–4]. The guidelines for the use of antiretroviral drugs in HIV-1-infected patients recommend that all patients must be screened for HBsAg before antiretroviral therapy (ART) is initiated, and that if a positive result is obtained, ART with tenofovir disoproxil fumarate (TDF) plus lamivudine (3TC) or emtricitabine must be initiated [5]. However, HBsAg testing is either not available or prohibitively expensive in several developing countries, and thus the HBV infection status is frequently unknown in the case of HIV-1-infected patients who have received a standard ART regimen including 3TC [6].

The drug 3TC is a cytidine analog that inhibits the reverse transcriptase (RT) of both HIV and HBV [7]. The major disadvantage associated with the use of this drug is that resistance mutations progressively emerge at an annual rate of 15–20% in HIV-1-HBV-coinfected patients in developed countries [8–11]. Consequently, new-generation RT inhibitors (RTIs) that feature comparatively higher resistance barriers have now been produced, and in the specific case of TDF, no resistance mutation has been detected in chronically HBV-infected patients after 3 years of therapy [12, 13]. However, in developing countries, most of these new drugs are not available or extremely expensive [14].

In China, government-supported ART including 3TC (ART-3TC) was started in 2003 [15], and numerous HIV-HBV-coinfected patients continue to remain on the ART-3TC regimen [16]. However, the effect of ART-3TC on HBV DNA loads and the prevalence of 3TC-resistant HBV mutants in HIV-HBV-coinfected patients have not been examined in China. Moreover, whether ART-3TC/TDF can reduce HBV DNA loads and the emergence of 3TC-resistant HBV mutants is unclear.

Here, we report the emergence of 3TC-resistance mutations in the HBV RT gene among HIV-HBV-coinfected Chinese patients during 96-week ART-3TC without TDF, and we describe the relationship between HBV DNA loads at baseline and the emergence of 3TC-resistant HBV.

## Materials and Methods

### Patients

**Fig 1:**
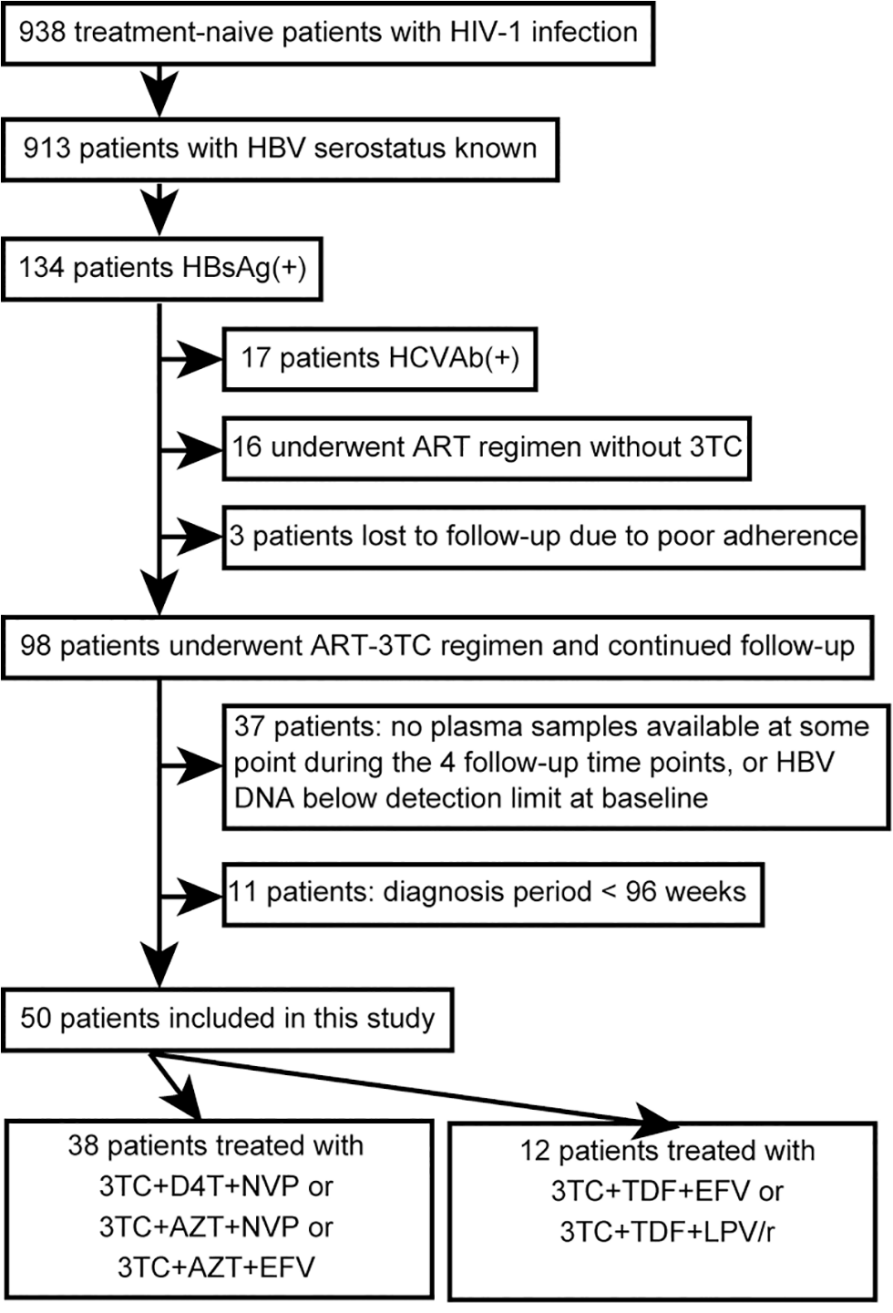
Flowchart depicting the selection of patients for this study.

This study was approved by the ethics committee at Peking Union Medical College Hospital (ClinicalTrials.gov Identifiers: NCT00618176 and NCT00872417). Patients were enrolled in Chinese cohort studies (National Key Technologies R&D Program for the 10th and 11th Five-year Plans) [16, 17] for examination of the response to the Chinese standard treatment regimen and the effectiveness of the treatment for HIV-1-infected patient. In these studies, patient selection and treatment methods were completely randomized. Informed consent was obtained from every participant. In China, patient plasma is collected at 12 centers (collaborative hospitals and local CDCs) that cover 23 provinces and cities (Beijing, Henan, Shaanxi, Shanghai, Fujian, Guangdong, Yunnan, Tianjin, Hebei, Neimenggu, Shandong, Liaoning, Jilin, Heilongjiang, Jiangsu, Zhejiang, Jiangxi, Shanxi, Anhui, Hunan, Hubei, Guangxi, and Sichuan). The patient plasma samples were collected between 2005 and 2014, and patients were classified according to the HBV-status criteria of the US Centers for Disease Control and Prevention [18]; these criteria have been used previously [16]. The flowchart in Fig 1 presents the patient-selection process, and Table 1 lists the clinical characteristics of the selected patients. We had access to stored patient blood samples collected before ART initiation (baseline) and after ART (12, 48, 96 weeks), and we selected 200 plasma samples from 50 patients who met previously described criteria [16]. Patients coinfected with hepatitis C virus were excluded from the analysis. The ART-3TC group comprised 38 patients who received treatment with 3TC/stavudine (d4T)/nevirapine (NVP) or 3TC/zidovudine (AZT)/NVP or 3TC/AZT/efavirenz (EFV) as first-line therapy, and the ART-3TC/TDF group included 12 patients who received 3TC/TDF/ritonavir-boosted lopinavir (LPV/r) or 3TC/TDF/EFV as first-line therapy (Table 1).

**Table 1:** Baseline demographic and clinical characteristics of 50 HIV-1-HBV-coinfected patients.

a. Clinical futures of patients					
	Subcategories			Value	
Gender (% of patients)	Male			44/50 (88.0%)	
	Female			6/50 (12.0%)	
Age, mean±SD (range, yr)				36.0 ± 9.5 (18–56)	
Routes of Transmission (% of patients)	MSM			22/50 (44.0%)	
	Heterosexal			21/50 (42.0%)	
	Blood transmission			1/50 (2.0%0)	
	Unknown			6/50 (12.0%)	
ART (number of patients)	ART-3TC	3TC+D4T+NVP or 3TC+AZT+NVP or 3TC+AZT+EFV		38	
	ART-3TC/TDF	TDF+3TC+EFV or TDF+3TC+LPV/r		12	
b.Viral loads and CD4 cell counts of patients					
		All (*n* = 50)	ART-3TC (*n* = 38)	ART-3TC/TDF (*n* = 12)	*P* value
Median HIV RNA loads [copies/mL (range)]	Before treatment	26993 (917–4.8×10^6^)	26205 (917–1.8 × 10^6^)	58972 (3395–4.8 × 10^6^)	> 0.05
	12wk	113 (< 20–4932)	113 (< 20–905)	124 (< 20–4932)	> 0.05
	48wk	< 20 (< 20–398)	< 20 (< 20–157)	< 20 (< 20–398)	> 0.05
	96wk	< 20 (< 20–99)	< 20 (< 20–99)	< 20	> 0.05
Median HBV DNA loads [IU/mL (range)]	Before treatment	7.8 × 10^5^ (< 20–2.5 × 10^8^)	3.4 × 10^6^ (< 20–1.7 × 10^8^)	2.7 × 10^5^ (399–2.5 × 10^8^)	> 0.05
	12wk	92 (< 20–8.2 × 10^7^)	724 (< 20–8.2 × 10^7^)	68 (< 20–5274)	> 0.05
	48wk	< 20 (< 20–8.1 × 10^4^)	< 20 (< 20–8.1 × 10^4^)	< 20 (< 20–53)	< 0.05
	96wk	< 20 (< 20–1.2 × 10^8^)	< 20 (< 20–1.2 × 108)	<20	< 0.05
Median CD4 cell counts [cells/mL (range)]	Before treatment	162 (2–343)	149 (3–343)	196 (2–332)	> 0.05
	12wk	297 (23–773)	259 (23–773)	320 (35–523)	> 0.05
	48wk	306 (53–705)	259 (53–616)	408 (87–705)	< 0.05
	96wk	337 (80–714)	307 (80–714)	456 (93–721)	< 0.05

### HBV markers and quantification of HBV DNA and HIV RNA

We tested for HBsAg and hepatitis virus e antigen (HBeAg) by performing automated chemiluminescent paramagnetic microparticle immunoassays on the Abbott Architect i2000SR system (Abbott Laboratories, Abbott Park, IL, USA). Levels of HBsAg (qHBsAg) were measured by performing quantitative enzyme immunoassays on the Abbott Architect i2000SR system, as per the manufacturer’s instructions. HBV DNA load was quantified using COBAS Ampliprep/COBAS TaqMan HBV Test, version 2.0. HIV RNA was quantified using COBAS Ampliprep/COBAS TaqMan HIV-1 Test, version 2.0 (Roche, Basel, Schweiz).

### Extraction and PCR-amplification of HBV DNA and HIV-1 RNA

HBV DNA was extracted from 200 μL of plasma by using the QIAamp DNA Blood Mini Kit (QIAGEN, Valencia, CA, USA). HIV-1 plasma RNA was extracted from 140 μL of plasma by using QIAamp Viral RNA Mini Kit (QIAGEN, Venlo, Netherlands). HBV drug-resistance mutations were detected by amplifying a partial coding region of HBV RT by means of nested PCR and then directly sequencing the products. For the first-round amplification of the HBV polymerase region, the following primer set was used: Pol/RT-1F: TTCCTGCTGGTGGCTCCAGTTC (nt 55–75); and Pol/RT-1R: GGAGTTCCGCAGTATGGATCGG (nt 1261–1282). The primers for the second-round amplification were the following: Pol/RT-2F: TGGATGTGTCTGCGGCGTTT (nt 361–393); and Pol/RT-2R-1: A or TCCCCATCTC or TTTTGTTTTGTG or TAGG (nt 838–861) and Pol/RT-2R-2: A or GCCCCAACGTTTGGTTTTATTAGG (nt 838–861). The sequences of these primers were designed based on the sequences archived in the HBV sequence database (http://hivdb.stanford.edu/HBV/HBVseq/development/HBVseq.html). The 501-bp PCR product (corresponding to nt 361–861 of the HBV RT coding sequence; NC_003977.1) was sequenced directly. This region has been reported to contain 10 amino acid residues that are closely related to 3TC resistance: rt169, rt173, rt180, rt181, rt184, rt194, rt202, rt204, rt233, and rt236 [9–11, 19–21]. These amino acids were identified because their sequences differ in a statistically significant manner from those in wild-type HBV RT [22–24]. The mutant sequences used for this study were obtained from the Stanford HBV RT drug-resistance database (http://hivdb.stanoford.edu/HBV/DB/cgi-bin/MutPrevByGenotypeRxHBV.cgi). The reference sequence of each HBV genotype from A to H was obtained from the NCBI/GenBank sequence database; the accession numbers are X02763, X51970, AF090842, AF297621, AM282986, D00329, AF100309, AB033554, AB073858, D00330, X04615, M12906, AB014381, AB033556, AB048704, X65259, M32138, X85254, J02203, X02496, X75657, AB032431, X69798, AB036910, AF223965, X75658, AF160501, AB064310, AF405706, AY090454, AY090457, and AY090460.

### Statistical analysis

For comparisons between 2 noncategorical variables, the Kruskal-Wallis test was used and the results are presented as medians and ranges. For comparisons between 2 categorical variables, the chi-square test or Fisher’s exact test was used. Statistical analyses were performed using GraphPad Prism 5.0 software (GraphPad Software Inc., San Diego, CA, USA).

## Results and Discussion

### Baseline characteristics

Of the 913 HIV infected patients who had enrolled in the national cohorts, 134 (14.7%) had tested HBsAg-positive, and 50 of these patients could be traced until 96 weeks of treatment. All 50 patients were Chinese, 88% of them were male, and the median age of this group was 36.0 years (range: 18–56 years) (Table 1). Most of the patients (86%) were infected through sexual contact, and 51.2% of these patients were men who have sex with men (“MSM”); 2% of the patients were infected through blood transfusion; and the remaining 12% were infected through unknown routes. This study included no intravenous drug users. The CD4+ T-cell count of the participants was <350/μL, and the median CD4+ T-cell count was 162/μL (range: 2–343 cells/μL). The median HIV RNA load was 26,993 copies/mL (range: 917–4.8 × 106 copies/mL), and the median HBV DNA load was 7.8 × 105 IU/mL (range: <20–2.5 × 108 IU/mL). The CD4+ T-cell counts, HIV-1 RNA loads, and HBV DNA loads did not differ between the ART-3TC and ART-3TC/TDF groups (Table 1).

The HBV RT coding region could be amplified from 46 out of 50 patient samples collected before ART initiation. A phylogenetic analysis performed using the PCR-product sequences indicated that 43.5% (20/46) of the patients were infected with HBV B genotype, 54.3% (25/46) with C genotype, and 2.2% (1/46) with D genotype (data not shown). Currently, the viral pathogenesis of HBV is considered to depend on the genotypes and not on geographic distributions [25–27]. HBV genotypes B and C are the major epidemic genotypes in China [28], and our study confirmed this. Genotype B is reported to be more responsive to 3TC than genotype C [29, 30], but we detected no large differences in the responsiveness of genotypes B and C to 3TC (S1 Fig). The crucial factor underlying treatment responsiveness was HBeAg in both genotypes: in our study, HBeAg-negative patients responded more strongly to the treatment than did HBeAg-positive patients (S1 Fig). The HBeAg positivity rate is also recognized to be high in HBV genotype C [31], but our results did not confirm this (S1 Table).

Because this study included 50 patients, we examined whether the sample size was appropriate for the statistics: we used Stata 11.0 software (StataCorp, College Station, TX, USA) and the “power and sample size analysis” method. In this analysis, we assumed that resistance rates were 50% in the ART-3TC group and 5% in the ART-3TC/TDF group after 2 years of treatment, based on previous reports [11, 20]. The calculation result showed that the statistical power was 0.90 (α = 0.05), because we enrolled 38 patients in the 3TC-treatment group and 12 patients in the TDF-treatment group. In this method, a statistical power of >0.80 is indicated to be statistically significant. Thus, we conclude that the number of patients analyzed can be considered to be statistically significant.

### Effect of ART including 3TC on HIV-1 and HBV replication

The median HIV-1 RNA loads of both the ART-3TC group and the ART-3TC/TDF group significantly decreased after treatment (P < 0.0001; Table 1), but HIV-1 viral-suppression efficacy did not differ between the treatments (P > 0.05; Table 1). The median CD4+ T-cell counts increased after both treatments (P < 0.0001; Table 1).

After treatment, the median HBV DNA loads of both groups were also significantly decreased (Table 1 and S2 Fig). At 48 weeks, the median HBV DNA load was below detectable levels, and at 96 weeks, the median HBV DNA load was still within the range considered “undetectable,” although the level now slightly increased in the ART-3TC group, indicating a possible HBV DNA rebound in certain patients. No such increase in the HBV DNA load was observed in the ART-3TC/TDF group (Table 1 and S2 Fig). In terms of individual cases, the HBV DNA loads of 16 and 15 patients were still detectable at 48 and 96 weeks, respectively. We suspect that the HIV ART was closely adhered to, because the HIV RNA load reduced in all patients. Measurement of the plasma concentrations of 3TC in patients in whom HBV breakthrough or insufficient suppression of HBV DNA loads was observed revealed that these concentrations were within the standard range in both groups of patients (data not shown).

### Emergence of 3TC-resistant HBV

We investigated the prevalence of 3TC-resistant mutants of the HBV RT gene by performing PCR-amplification and directly sequencing the PCR products; the results are presented in Table 2. The RT coding region was amplified from the samples of the 38 patients in the ART-3TC (without TDF) group: 34 plasma samples (89.5%) at baseline, 11 samples (28.9%) after 12 weeks of treatment, 6 (15.8%) after 48 weeks, and 10 (26.3%) after 96 weeks. Moreover, the RT coding region was PCR-amplified from the samples of the 12 patients in the ART-3TC/TDF group: 12 samples (100%) at baseline, and 3 samples (25%) after 12 weeks of treatment. However, we detected no PCR product in the patient samples after 48 weeks of treatment.

**Table 2:** Emergence of lamivudine-resistant HBV in long-term ART-3TC.

a. PCR success rate and drug resistant mutation rate					
	ART	0 wk	12 wk	48 wk	96 wk
PCR success rate	ART-3TC	89.5% (34/38)	28.9% (11/38)	15.8% (6/38)	26.3% (10/38)
	ART-3TC/TDF	100% (12/12)	25.0% (3/12)	0% (0/12)	0% (0/12)
Drug resistance rrelated mutation rate	ART-3TC	0% (0/34)	0% (0/11)	16.7% (1/6)	80% (8/10)
	ART-3TC/TDF	0% (0/12)	0% (0/3)	ND	ND
b. The details of mutants					
ART	mutation site(s)	Case number			
		0 wk	12 wk	48 wk	96 wk
ART-3TC	L180M	0	0	0	0
	M204I/V	0	0	1	2
	L180M, M204I/V	0	0	0	5
	T184S, M204I/V	0	0	0	1
ART-3TC/TDF	L180M	0	0	ND	ND
	M204I/V	0	0	ND	ND
	L180M, M204I/V	0	0	ND	ND
	T184S, M204I/V	0	0	ND	ND

The results of sequence analysis performed for amino acid positions 180 (Leu) and 204 (Met) demonstrated that long-term treatment under the ART-3TC regimen (without TDF) resulted in the emergence of the 3TC-resistance mutations: None of the patients showed any mutation at 180L and 204M at baseline. We detected single amino acid mutations at position 204 in 2 cases, double amino acid mutations at positions 180 and 204 in 5 cases, and mutations at positions 184 and 204 in a single case (Table 2). The main mutation sites identified here agree with previously published data [9–11, 20, 21]. However, the mutation rate recorded after 96 weeks of treatment, 23.5% (8/34 patients), was lower than that in Western countries, 50% [20]. This difference could depend on the distinct environments related to the HBV infection routes: In China, infection occurs mainly through vertical infection, which differs from the lateral infection prevalent in Western countries.

ART-3TC/TDF was administered to fewer patients (12 cases) than was ART-3TC (38 cases); however, the addition of TDF appeared to have effectively suppressed HBV DNA loads in 96 weeks (Table 2). Because the HBV RT coding region could not be PCR-amplified after ART-3TC/TDF, we were unable to investigate whether mutations related to TDF or 3TC resistance would appear in the ART-3TC/TDF cases after treatment for >96 weeks. However, no resistance mutation in the HBV RT coding region has been reported in patients chronically infected with HBV even after 3 years of TDF therapy [12, 13]. Thus, the HBV antigen test is recommended for the identification of HBV-coinfected patients and the use of TDF would be favorable for HIV-HBV-coinfected patients. The new treatment guideline for HIV-1 patients was issued by the Chinese Ministry of Health [32]. Although TDF is now available in China in accordance with this new guideline, no long-term follow-up study has previously been conducted in China to evaluate HBV drug resistance in the setting of HIV coinfection in Chinese patients. Thus, this is the first report of a long-term follow-up study of TDF treatment used for HIV-1-HBV-coinfected patients in China.

**Fig 2:**
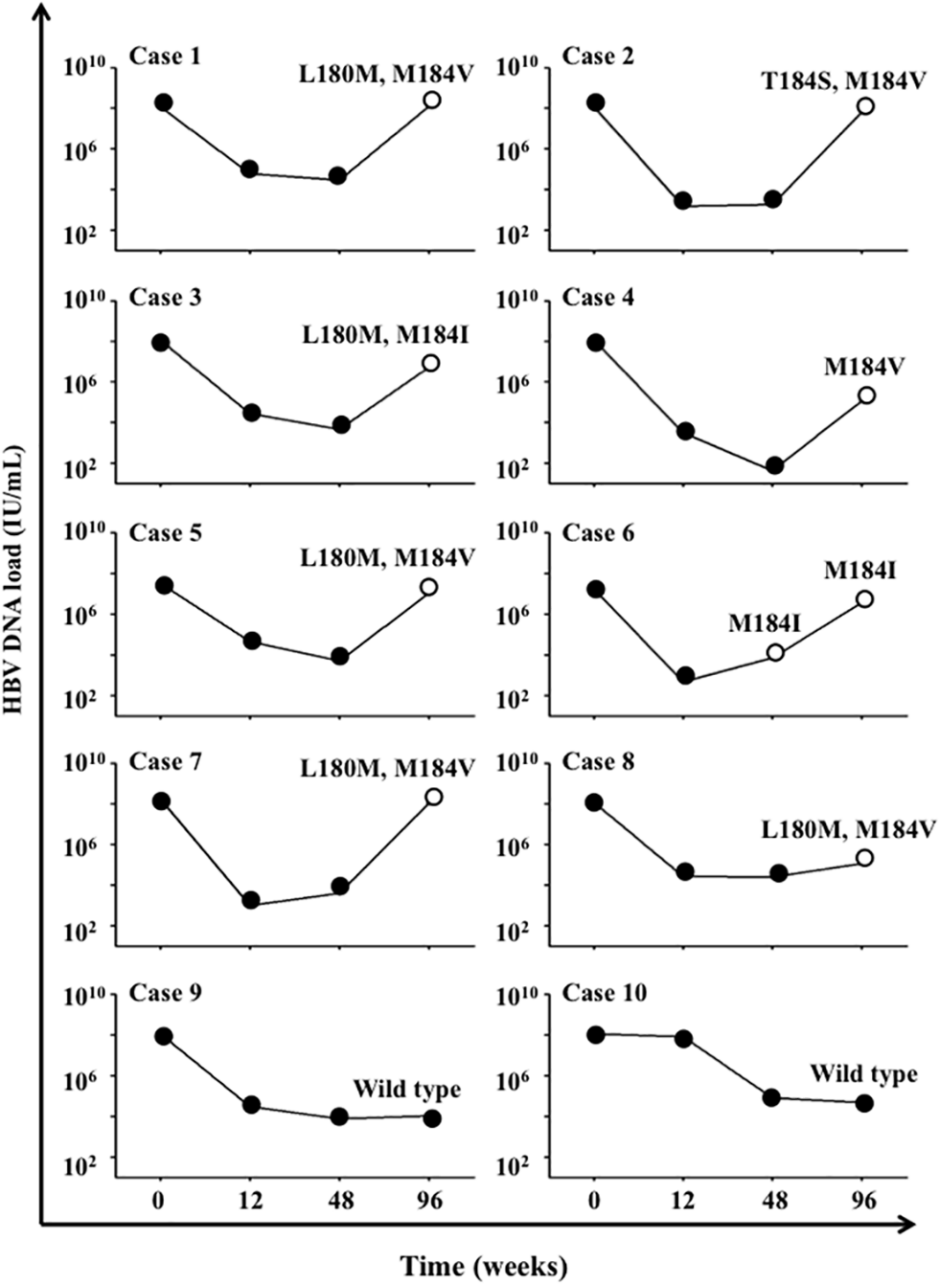
Changes in HBV DNA loads during ART in patients who exhibited insufficient suppression of HBV DNA loads. The case numbers 1–10 represent the 10 patients in whom HBV DNA loads were insufficiently suppressed. Mutations related to 3TC resistance were detected in samples collected from No.1–No.8 but not No.9 and No.10. The open circles indicate the time points at which the mutations were detected, and the mutated amino acids and their sequence numbers are listed above these circles.

**Fig 3:**
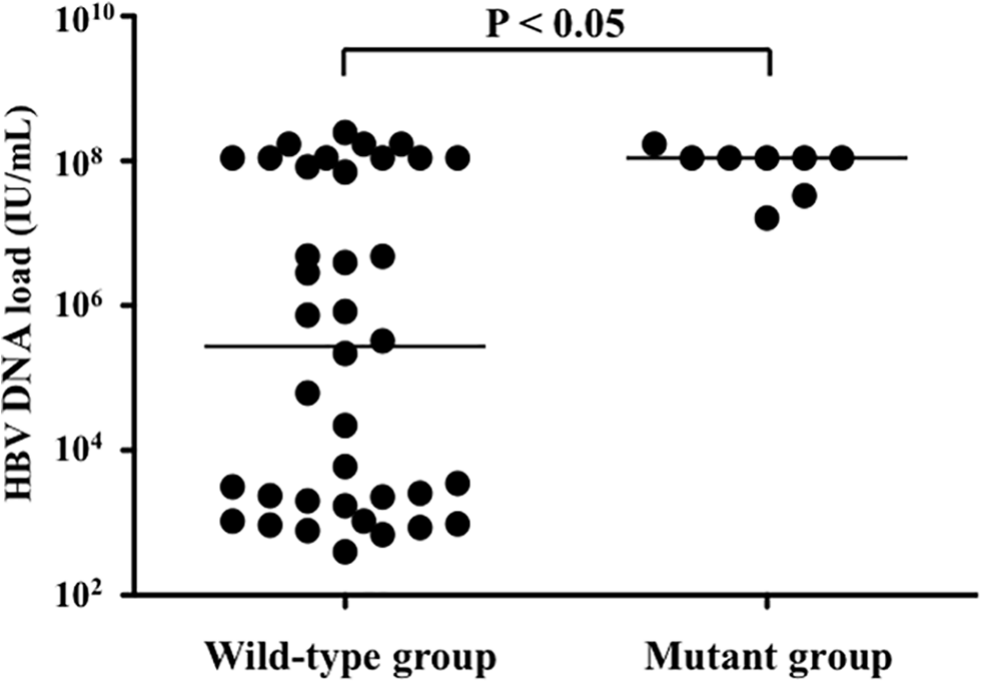
Correlation between baseline HBV DNA loads and emergence of 3TC-resistance mutations. Each dot represents the HBV DNA load of a clinical sample at baseline. The horizontal lines indicate the median values of HBV DNA loads: 2.7 × 105 IU/mL in the wild-type group, 1.1 × 108 IU/mL in the group in which 3TC-resistance mutations emerged (mutant group).

Intriguingly, HBV breakthrough or insufficient suppression of HBV DNA load was observed in 10 patients of the ART-3TC group after 96-week treatment, and in 8 of these patients, mutations associated with 3TC resistance were detected (Table 2, Fig 2 and S3 Fig). In the patients harboring these mutants, DNA loads at baseline were significantly higher than those in the remaining patients (P < 0.05; Fig 3). However, with regard to the emergence rate of 3TC-resistance mutations, we detected no significant differences between genotypes B and C (S1 Fig). In this study, 16 patients (32%) were HBeAg-positive, and the HBV DNA load of these patients was significantly higher than that of HBeAg-negative patients (P < 0.0001; S4 Fig). In both cases, the HBV DNA load was markedly reduced after ART with or without the inclusion of TDF (S4 Fig). However, in the patient group in which the 3TC mutants emerged, both ART-3TC and ART-3TC/TDF did not effectively suppress HBV DNA loads (S4 Fig). Furthermore, in the HBeAg-positive patients, ART-3TC suppressed HBV DNA loads significantly more weakly than did ART-3TC/TDF (P < 0.0001; S4 Fig). Recent clinical studies on chronic hepatitis B have revealed that high HBV DNA loads are correlated with HBeAg positivity, which plays a crucial role in the progression of hepatitis [20, 33–35]. Thus, our data also strongly suggest that HBV DNA loads must be measured and, if possible, HBeAg tests must be conducted in order to check for and avoid the emergence of 3TC-resistant HBV. We could not analyze a large number of patients for whom a TDF-based regimen has been used, because treatments that include TDF have only recently become available for HIV patients in China. However, we used Stata 11.0 software and calculated the statistical power based on previous data obtained in the case of HIV-HBV coinfection, and our results showed that the statistical power here reached 0.90. This indicates that the number of patients included in our analysis yielded statistically significant data.

## Conclusions

Our findings have clearly demonstrated that ART-3TC is associated with the emergence of 3TC-resistant HBV in patients coinfected with HIV-1 and HBV. The emergence of 3TC-resistance mutations was closely associated with high HBV DNA loads. To avoid the emergence of 3TC-resistant HBV, the monitoring of HBV DNA loads and the use of ART including TDF are recommended for patients coinfected with HIV and HBV.

## Supporting Information

S1 Fig Correlation between HBV genotypes and HBV DNA loads and relationship with HBeAg. (A) Correlation between HBV genotypes B and C and viral DNA loads. Open columns and gray columns indicate the HBV DNA loads after 0 and 96 weeks, respectively, of treatment in HBV genotype B (n = 20) patients and genotype C (n = 25) patients. HBV DNA loads did not differ significantly between groups infected with HBV genotypes B and C at baseline (P > 0.05; median HBV DNA loads: genotype B, 4.4 × 106 IU/mL; genotype C, 7.4 × 105 IU/mL) and after 96-week treatment (median HBV DNA loads: <20 IU/mL in both genotypes). (B & C) Correlation between HBeAg status and HBV DNA loads. (B) In the group infected with HBV genotype B, 12 patients were HBeAg-negative [(HBeAg(-); open columns] and 8 were HBeAg-positive [HBeAg(+); gray columns]. The HBV DNA load in HBeAg(+) patients was significantly higher than that in HBeAg(-) patients at baseline [P < 0.05; median HBV DNA loads: HBeAg(-), 4737 IU/mL; HBeAg(+), 1.1 × 108 IU/mL] and after 96-week treatment [P < 0.0001; median HBV DNA loads: HBeAg(-), <20 IU/mL; HBeAg(+), 6.0 × 104 IU/mL]. (C) In the group infected with HBV genotype C, 17 patients were HBeAg(-) (open columns) and 8 were HBeAg(+) (gray columns). The HBV DNA load of HBeAg(+) patients was significantly higher than that of HBeAg(-) patients at baseline [P < 0.001; median HBV DNA loads: HBeAg(-), 2361 IU/mL; HBeAg(+), 1.4 × 108 IU/mL] and after 96-week treatment [P < 0.05; median HBV DNA loads: HBeAg(-), <20 IU/mL; HBeAg(+), 2040 IU/mL]. (D & E) Changes in HBV DNA loads during ART in patients infected with HBV genotype B (D) and C (E). The 3TC-resistance mutations were detected in 4 cases in both genotype B and genotype C; the mutation-detection time points are indicated by open diamonds. (F & G) Correlation between ART regimen (ART-3TC) and emergence of 3TC-resistant mutants in genotypes B and C. In the genotype B group (F), 14 patients harbored wild-type HBV during the treatment period (wild-type group) and 4 patients harbored 3TC-resistant HBV mutants after 96 weeks of treatment (mutant group). In the genotype C group (G), 11 patients harbored wild-type HBV (wild-type group) and 4 patients harbored 3TC-resistant HBV mutants after 96 weeks (mutant group). In the case of both genotypes, the median HBV DNA loads at baseline did not differ significantly between the wild-type and mutant groups (P > 0.05), but the DNA load decreased markedly after the 96-week treatment in the wild-type group (P < 0.05). However, we did not detect any suppressive effect of the treatments in the mutant group (P > 0.05). Error bars indicate the interquartile range. The statistical significance of differences was calculated using the Mann-Whitney test.
(TIF)

S2 Fig Changes in HBV DNA loads in the 2 ART groups, ART-3TC and ART-3TC/TDF. The left and right panels show the changes in the HBV DNA load of the patients during ART-3TC and ART-3TC/TDF, respectively. Boxes indicate the interquartile range and the internal bars indicate the medians of the HBV DNA loads. Error bars indicate maximal and minimal values of HBV DNA. After the treatment, the HBV DNA load was lowered significantly in both treatment groups (ART-3TC: P < 0.0001; ART-3TC/TDF: P < 0.05). The statistical analysis also revealed that HBV DNA loads after ART-3TC/TDF were significantly lower than those after ART-3TC (P < 0.0001).
(TIF)

S3 Fig Changes in HBV DNA loads during ART in patients in whom the treatment was successful. The figure shows the changes in the HBV DNA load of each patient during ART.
(TIF)

S4 Fig Correlation between HBeAg status and HBV DNA loads. (A) Correlation between HBeAg status and HBV DNA loads: open columns indicate the HBV DNA loads at baseline and after 96-week treatment in HBeAg-negative patients [HBeAg(-); n = 34]; gray columns indicate the corresponding loads in HBeAg-positive patients [HBeAg(+); n = 16]. The HBV DNA load of HBeAg(+) patients was significantly higher than that of HBeAg(-) patients at baseline [P < 0.0001; median HBV DNA loads: HBeAg(-), 2845 IU/mL; HBeAg(+), 1.1 × 108 IU/mL] and after 96-week treatment [median HBV DNA loads: HBeAg(-), <20 IU/mL (below detection level); HBeAg(+), 8226 IU/mL]. (B & C) Correlation between ART regimen and HBV DNA loads, shown for each HBeAg status. In the HBeAg(-) group (B), 25 and 9 patients received ART-3TC (open columns) and ART-3TC/TDF (gray columns), respectively; the HBV DNA loads did not differ significantly between patients who received these 2 treatments [median HBV DNA loads at baseline (0 week): 2559 IU/mL in ART-3TC patients and 3130 IU/mL in ART-3TC/TDF patients; median viral DNA loads after 96 weeks: <20 IU/mL in both treatment groups]. In the HBeAg(+) group (C), 13 and 3 patients received ART-3TC (open columns) and ART-3TC/TDF (gray columns), and their median HBV DNA levels were almost same at baseline (1.1 × 108 IU/mL in ART-3TC patients and 1.7 × 108 IU/mL in ART-3TC/TDF patients). However, the median HBV DNA load of the ART-3TC group was significantly higher than that of the ART-3TC/TDF group after 96-week treatment (P < 0.0001; ART-3TC, 4.8 × 104 IU/mL; ART-3TC/TDF, <20 IU/mL). These results suggest that the ART regimen including TDF was more effective for HBeAg(+) patients than for HbeAg(-) patients. (D & E) Correlation between ART regimen (ART-3TC) and the emergence of 3TC-resistant mutants, shown for each HBeAg status. In the HBeAg(-) group (D), 23 patients (wild-type group) harbored wild-type HBV and 2 patients (mutant group) harbored 3TC-resistant HBV mutants at 96 weeks. The median HBV DNA load of the mutant group at baseline (6.3 × 107 IU/mL) was higher than that of the wild-type group (2361 IU/mL). The median HBV DNA load of the wild-type group decreased significantly after 96 weeks of ART (P < 0.0001), but the treatment produced no suppressive effect in the mutant group; we could not perform statistical analysis because the mutant group included only 2 patients. In the HBeAg(+) group (E), 7 patients (wild-type group) harbored wild-type HBV and 6 patients (mutant group) harbored 3TC-resistant HBV mutants at 96 weeks. The median HBV DNA loads at baseline were almost the same in the wild-type and mutant groups (P > 0.05; 1.1 × 108 IU/mL). The median HBV DNA load of the wild-type group decreased significantly after treatment (P < 0.0001; 3073 IU/mL at 96 weeks). However, we detected no suppressive effect of ART in the mutant group (P > 0.05; 3.6 × 107 IU/mL at 96 weeks). The quantification is described in Materials and Methods. The error bars indicate the interquartile range. The statistical significance of differences was calculated using the Mann-Whitney test.
(TIF)

S1 Table HBV genotype correlation with HBeAg positivity and mutant rate. (DOCX)
