# Supplementary figures and images for: Emergence of Lamivudine-Resistant HBV during Antiretroviral Therapy Including Lamivudine for Patients Coinfected with HIV and HBV in China
Source: PLoS One. 2015 Aug 19;10(8):e0134539. doi: 10.1371/journal.pone.0134539 (PMC4543549; doi:10.1371/journal.pone.0134539)

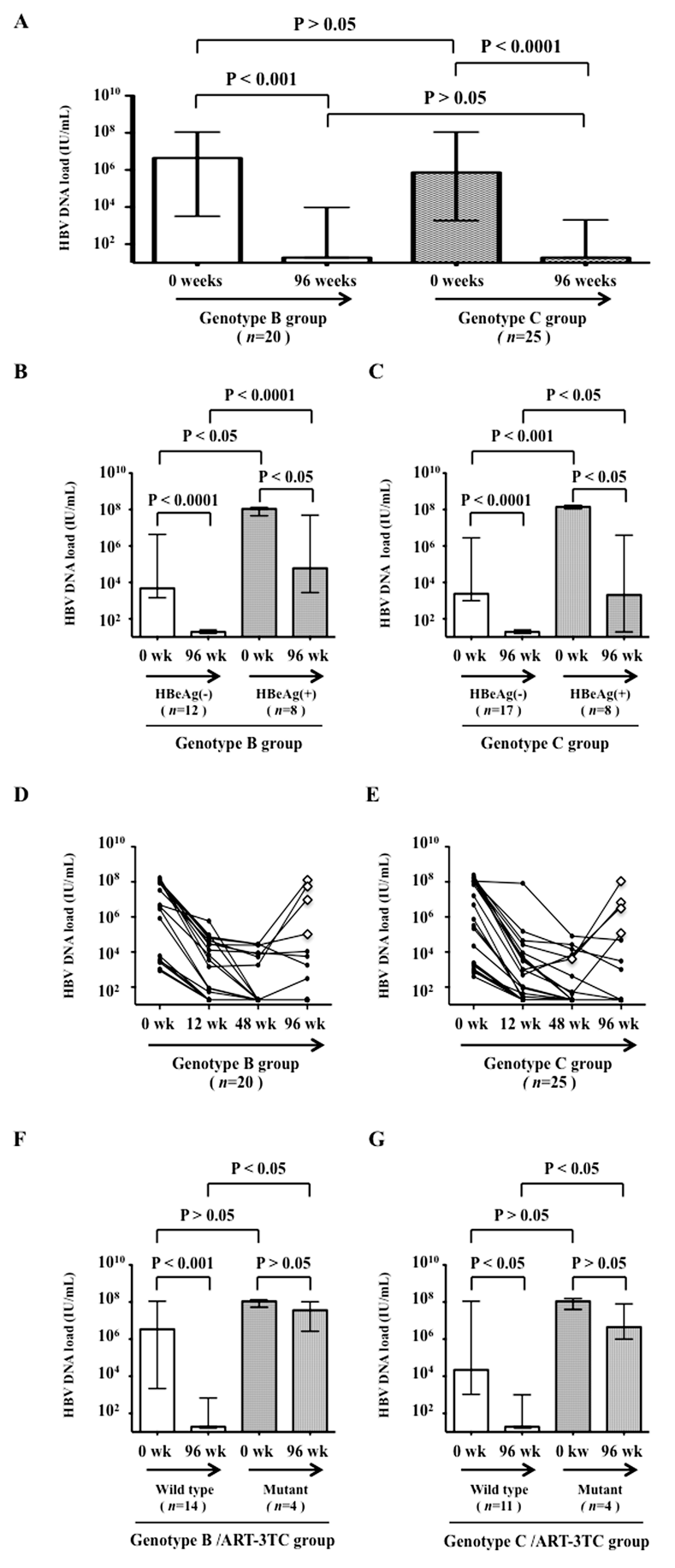

Supplement: S1 Fig — (A) Correlation between HBV genotypes B and C and viral DNA loads. Open columns and gray columns indicate the HBV DNA loads after 0 and 96 weeks, respectively, of treatment in HBV genotype B (n = 20) patients and genotype C (n = 25) patients. HBV DNA loads did not differ significantly between groups infected with HBV genotypes B and C at baseline (P > 0.05; median HBV DNA loads: genotype B, 4.4 × 10^6^ IU/mL; genotype C, 7.4 × 10^5^ IU/mL) and after 96-week treatment (median HBV DNA loads: <20 IU/mL in both genotypes). (B & C) Correlation between HBeAg status and HBV DNA loads. (B) In the group infected with HBV genotype B, 12 patients were HBeAg-negative [(HBeAg(-); open columns] and 8 were HBeAg-positive [HBeAg(+); gray columns]. The HBV DNA load in HBeAg(+) patients was significantly higher than that in HBeAg(-) patients at baseline [P < 0.05; median HBV DNA loads: HBeAg(-), 4737 IU/mL; HBeAg(+), 1.1 × 10^8^ IU/mL] and after 96-week treatment [P < 0.0001; median HBV DNA loads: HBeAg(-), <20 IU/mL; HBeAg(+), 6.0 × 10^4^ IU/mL]. (C) In the group infected with HBV genotype C, 17 patients were HBeAg(-) (open columns) and 8 were HBeAg(+) (gray columns). The HBV DNA load of HBeAg(+) patients was significantly higher than that of HBeAg(-) patients at baseline [P < 0.001; median HBV DNA loads: HBeAg(-), 2361 IU/mL; HBeAg(+), 1.4 × 10^8^ IU/mL] and after 96-week treatment [P < 0.05; median HBV DNA loads: HBeAg(-), <20 IU/mL; HBeAg(+), 2040 IU/mL]. (D & E) Changes in HBV DNA loads during ART in patients infected with HBV genotype B (D) and C (E). The 3TC-resistance mutations were detected in 4 cases in both genotype B and genotype C; the mutation-detection time points are indicated by open diamonds. (F & G) Correlation between ART regimen (ART-3TC) and emergence of 3TC-resistant mutants in genotypes B and C. In the genotype B group (F), 14 patients harbored wild-type HBV during the treatment period (wild-type group) and 4 patients harbored 3TC-resistant HBV mutants after 96 weeks of [file pone.0134539.s001.tif]

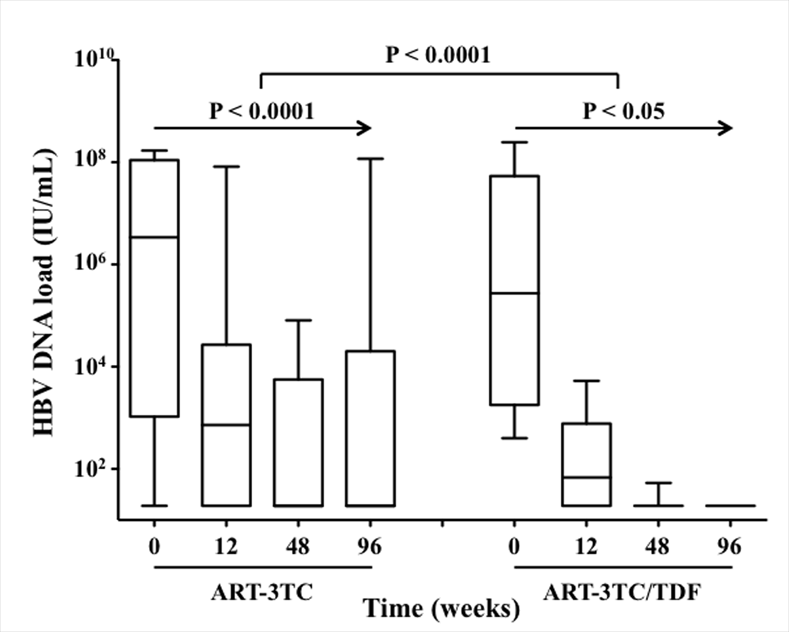

Supplement: S2 Fig — The left and right panels show the changes in the HBV DNA load of the patients during ART-3TC and ART-3TC/TDF, respectively. Boxes indicate the interquartile range and the internal bars indicate the medians of the HBV DNA loads. Error bars indicate maximal and minimal values of HBV DNA. After the treatment, the HBV DNA load was lowered significantly in both treatment groups (ART-3TC: P < 0.0001; ART-3TC/TDF: P < 0.05). The statistical analysis also revealed that HBV DNA loads after ART-3TC/TDF were significantly lower than those after ART-3TC (P < 0.0001). (TIF) [file pone.0134539.s002.tif]

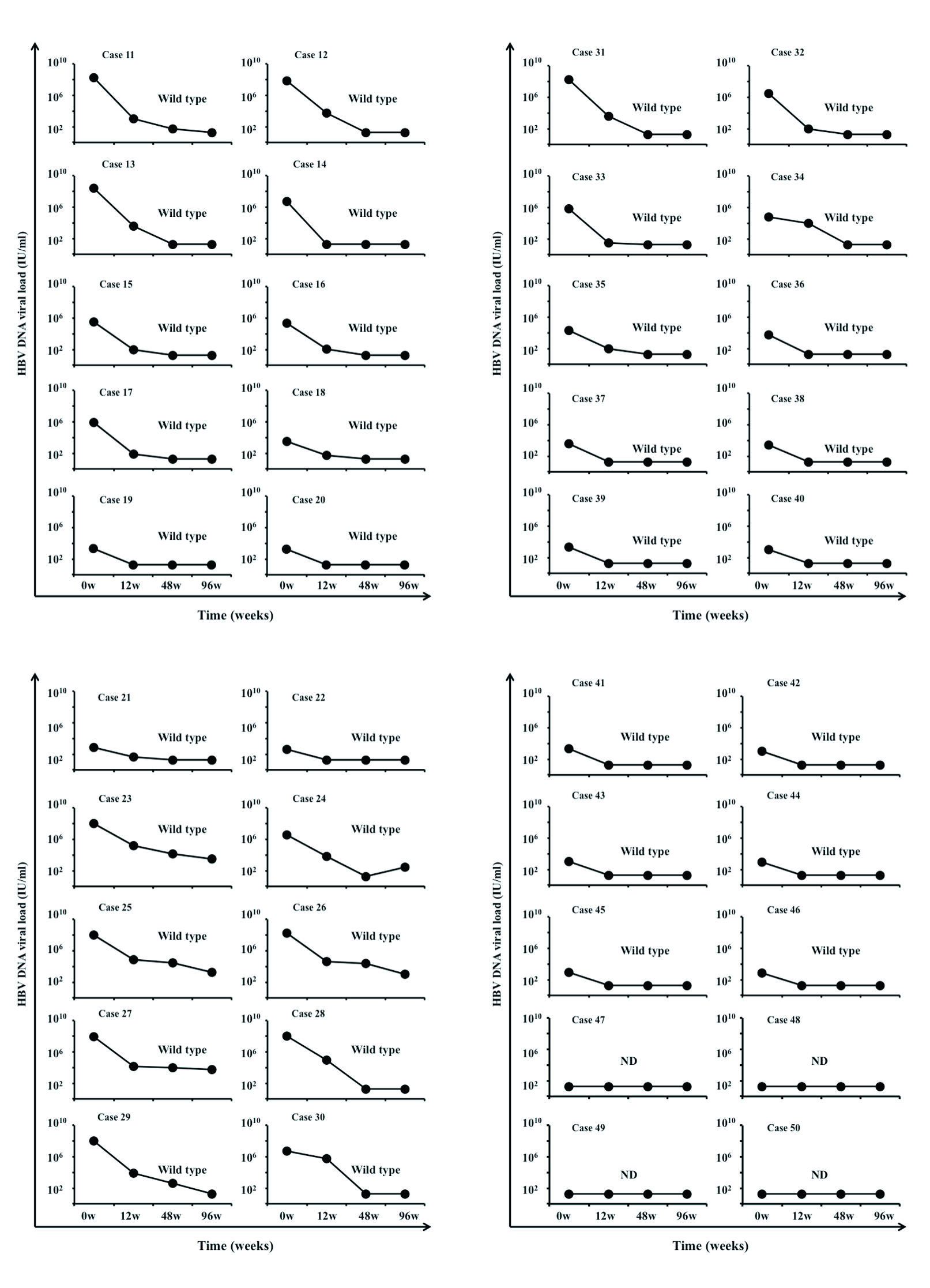

Supplement: S3 Fig — The figure shows the changes in the HBV DNA load of each patient during ART. (TIF) [file pone.0134539.s003.tif]

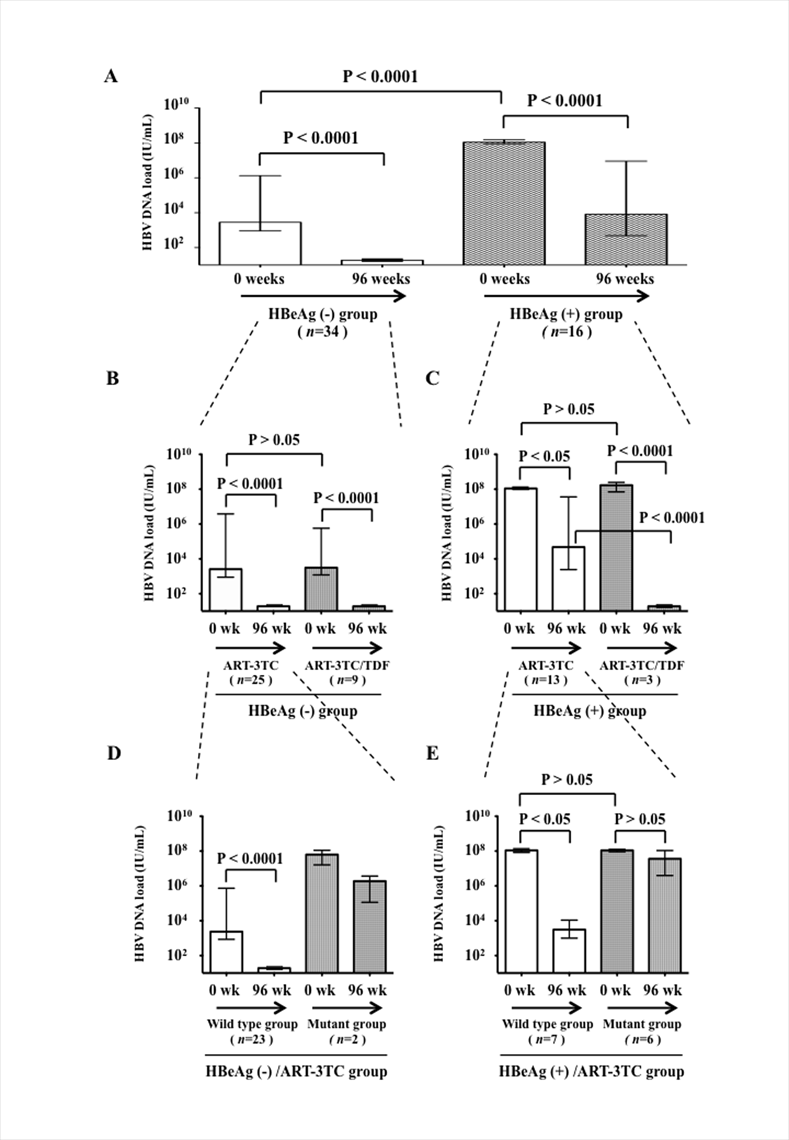

Supplement: S4 Fig — (A) Correlation between HBeAg status and HBV DNA loads: open columns indicate the HBV DNA loads at baseline and after 96-week treatment in HBeAg-negative patients [HBeAg(-); n = 34]; gray columns indicate the corresponding loads in HBeAg-positive patients [HBeAg(+); n = 16]. The HBV DNA load of HBeAg(+) patients was significantly higher than that of HBeAg(-) patients at baseline [P < 0.0001; median HBV DNA loads: HBeAg(-), 2845 IU/mL; HBeAg(+), 1.1 × 10^8^ IU/mL] and after 96-week treatment [median HBV DNA loads: HBeAg(-), <20 IU/mL (below detection level); HBeAg(+), 8226 IU/mL]. (B & C) Correlation between ART regimen and HBV DNA loads, shown for each HBeAg status. In the HBeAg(-) group (B), 25 and 9 patients received ART-3TC (open columns) and ART-3TC/TDF (gray columns), respectively; the HBV DNA loads did not differ significantly between patients who received these 2 treatments [median HBV DNA loads at baseline (0 week): 2559 IU/mL in ART-3TC patients and 3130 IU/mL in ART-3TC/TDF patients; median viral DNA loads after 96 weeks: <20 IU/mL in both treatment groups]. In the HBeAg(+) group (C), 13 and 3 patients received ART-3TC (open columns) and ART-3TC/TDF (gray columns), and their median HBV DNA levels were almost same at baseline (1.1 × 10^8^ IU/mL in ART-3TC patients and 1.7 × 10^8^ IU/mL in ART-3TC/TDF patients). However, the median HBV DNA load of the ART-3TC group was significantly higher than that of the ART-3TC/TDF group after 96-week treatment (P < 0.0001; ART-3TC, 4.8 × 10^4^ IU/mL; ART-3TC/TDF, <20 IU/mL). These results suggest that the ART regimen including TDF was more effective for HBeAg(+) patients than for HbeAg(-) patients. (D & E) Correlation between ART regimen (ART-3TC) and the emergence of 3TC-resistant mutants, shown for each HBeAg status. In the HBeAg(-) group (D), 23 patients (wild-type group) harbored wild-type HBV and 2 patients (mutant group) harbored 3TC-resistant HBV mutants at 96 weeks. The median HBV DNA load of the mutant group at baseline [file pone.0134539.s004.tif]
