# Supplementary material for: Emergence of Lamivudine-Resistant HBV during Antiretroviral Therapy Including Lamivudine for Patients Coinfected with HIV and HBV in China
Source: PLoS One. 2015 Aug 19;10(8):e0134539. doi: 10.1371/journal.pone.0134539 (PMC4543549; doi:10.1371/journal.pone.0134539)
Supplement: S1 Table — (DOCX) [file pone.0134539.s005.docx]

**Supplementary Table 1. HBV genotype correlation with HBeAg positivity and mutant rate.**

|  | Total patient number | HBeAg(-) | HBeAg(+) | *P* valu | Wild type | Mutant | *P* valu |
| --- | --- | --- | --- | --- | --- | --- | --- |
| Genotype B | 20 | 12(60%) | 8(40%) | P > 0.05 | 16(80%) | 4(20%) | P > 0.05 |
| Genotype C | 25 | 17(66%) | 8(32%) |  | 21(84%) | 4(16%) |  |
